# Study on the Equation of State and Jet Forming of 3D-Printed PLA and PLA-Cu Materials

**DOI:** 10.3390/polym15173564

**Published:** 2023-08-28

**Authors:** Jianya Yi, Ruijie Hao, Qing Ji, Siman Guan, Zhijun Wang, Jianping Yin

**Affiliations:** 1 School of Mechatronic Engineering, North University of China, Taiyuan 030051, China; hrj2021315@163.com (R.H.); gsm2021315@163.com (S.G.); wzj@nuc.edu.cn (Z.W.); yjp123@nuc.edu.cn (J.Y.); 2 Northwest Institute of Mechanical & Electrical Engineering, Xianyang 712099, China; sxwn_jiqing@163.com

**Keywords:** liner, PLA, PLA-Cu, equation of state, jet formation

## Abstract

In order to improve the research and development efficiency and quality of low-density liners in production and scientific research development, PLA and PLA-Cu composite liners were prepared based on 3D-printing technology. In this paper, the relationship between the shock wave velocity *D* and the particle velocity *u* of PLA and PLA-Cu materials was tested by a one-stage light gas gun experiment device, and then the Grüneisen equation of state parameters of the two materials was obtained by fitting. The forming process of the two jets was numerically simulated by using the equation of state. When combined with the pulsed X-ray shooting results of the jets, it was found that the jets of the two materials showed obvious characteristics of “expansion particle flow”, and the head of the PLA jet had a gasification phenomenon. The length of the PLA jet at 20 μs in the numerical simulation was 127.2 mm, and the average length of the PLA jet at 20 μs in the pulsed X-ray shooting experiment was 100.45 mm. The length of the PLA jet gasification part accounted for about 21% of the total length of the jet. The average velocity of the head of the PLA jet is 7798.35 m/s, and the average velocity of the head of the PLA-Cu jet is 8104.25 m/s. In this paper, 3D-printing technology is used to prepare the liner for the first time, aiming to open up a new preparation technology and provide a new material selection for low-density material liners.

## 1. Introduction

With the continuous updating of armor protection technology, anti-armor weapons are also developing continuously, and the research of non-metallic material liners has gradually become a hot spot. Non-metallic materials for liners are divided into inorganic non-metals (including glass [1] and ceramics [2]) and organic non-metals (various polymer materials [3,4,5,6]). At present, the processing technology of low-density liners is mainly traditional turning, cold-pressing sintering, hot-pressing, twin screw extrusion and other processing technologies, which have the disadvantages of high cost, low processing accuracy, and efficiency. As a core technology in the fourth-generation industrial revolution, 3D-printing technology is based on the advanced design and intelligent manufacturing of “additive manufacturing” thinking. It can effectively control the printing accuracy while reducing the cumbersome processing steps of the traditional processing technology to meet the requirements of rapid prototyping. Therefore, it is suitable for the preparation of high-precision liners. It has become possible to produce composite polymers by doping powder materials heterogeneously into a polymer matrix in order to produce parts of higher subsequent mechanical strength through the 3D-printing process. The technique is capable of building parts of any complicated geometry in the least possible time without incurring extra costs due to the absence of tooling and with minimal material waste. In reference [7], related research on the preparation of liners by 3D printing was carried out, but the forming characteristics of the jet were not described in detail. Therefore, the forming characteristics of polymer jets were studied in detail in this paper.

Three-dimensional-printing technology is also called “Additive Manufacturing (AM)” technology. It takes fine materials as the starting point and digital control as the means to creatively realize the simultaneous preparation of materials while manufacturing parts. Additive manufacturing is a method of manufacturing solid parts by layer-by-layer accumulation of materials. Fused deposition modeling (FDM) is one of the molding processes of 3D-printing technology. The 3D printer using FDM printing technology is most suitable for mass production in terms of cost, efficiency and feasibility. Therefore, this paper chooses FDM molding technology suitable for thermoplastic materials to complete the preparation of the liner. The basic principle of FDM molding is as follows: Firstly, the filamentary hot-melt material is heated in the nozzle to present a molten state, and the computer program is used to control the printer head to selectively apply the material to the specified position of the workbench. The molten material is rapidly solidified into a layer cross-section after being squeezed out by the nozzle. After the previous layer is formed, the support platform of the printer drops a certain height according to the preset layer height, and the nozzle continues to print the next layer on the basis of the previous layer until the entire solid model is formed [8,9].

Polylactic acid (PLA) is a thermoplastic polyester. Because of its good biodegradability, biocompatibility and no toxic side effects, PLA has become the most commonly used polymer material for 3D printing. PLA material is widely used in the field of 3D printing, and its material itself has many advantages, so in this paper, PLA was the chosen material for the front liner of the tandem warhead to carry out research. At the same time, in order to enhance the forming performance of the jet formed by the pure polymer liner, the penetration performance of the copper powder-filled PLA composite liner (PLA-Cu) was also compared. Because the filamentary 3D-printing material is processed by FDM technology, its mechanical properties are not only related to the characteristics of the material itself but also affected by the characteristics of the 3D printer, process parameters and post-processing. Therefore, before studying the jet performance of 3D-printed PLA and PLA-Cu liners, it is necessary to study the impact mechanical properties of the two materials.

Ji Qing et al. [10] obtained the stress–strain curves of polylactic acid (PLA) materials and copper powder-filled polylactic acid (PLA-Cu) materials at different strain rates by using the universal material testing machine and the split Hopkinson pressure bar test device. Chacón, J. [11] described the effects of different construction directions, printing layer thickness and nozzle extrusion speed on the mechanical properties of 3D-printed PLA parts under tensile and bending tests. Verbeeten W.M.H. [12] studied the dependence of the yield stress of 3D-printed PLA parts on the strain rate under anisotropic conditions. The sample with a filling angle of 0° (horizontal) has a stronger strain rate dependence than the 90°(vertical) sample, and the strain rate dependence of the tensile sample is stronger than that of the compressive sample. Vidakis N. [13] et al. carried out tensile tests of PLA samples at various strain rates. The results showed that the tensile strength of PLA samples gradually increased with the increase in strain rate, and the brittleness of PLA samples was also found to be high. In the study of filling density, Ambati S.S. [14] found that the tensile strength of PLA samples is proportional to the increase in density. Farazin A. and Mohammadimehr M. [15] found that with the increase in filling density, although the tensile strength of the parts increased, the brittleness of the samples also increased. Samykano M. [16] also found that the filling density has a strong influence on the elastic modulus, fracture strain and toughness of the parts. Kuznetsov et al. [17] believed that changing the deposition temperature between the polymer layers has a significant positive effect on the viscosity between each layer, which can make the strength of the interlayer adhesive similar to the strength of the embryo. Jayanth N. [18] attributed the poor mechanical properties of 3D-printed parts to the poor adhesion between the layers and the residual stress caused by the temperature difference. At the same time, they proposed that the heat treatment method can significantly improve the internal stress and the adhesion between the filaments in the printing process and ultimately improve the mechanical properties of the parts. Bayraktar Ö [19] studied the effects of nozzle temperature, layer thickness and printing direction on the tensile properties of PLA. The results showed that the tensile strength of PLA material was the highest when the printer was crossed (+45°/−45°). It was also found that the lower nozzle temperature was not conducive to the diffusion of polymer materials in the solution, and a large number of cross-linked molecular bonds could not be formed, which would reduce the strength of the tensile sample and the increase in layer thickness would reduce the tensile strength. Khosravani M. R et al. [20] showed that parts printed in the horizontal direction have higher strength than those printed in the vertical direction. Hsueh M.H. [21] et al. obtained similar results when studying the effect of nozzle temperature.

In addition to PLA materials, some scholars also studied the additive manufacturing technology of other materials. Rahmatabadi Davood et al. [22] studied the influence of fused deposition modeling (FDM) process parameters on the shape memory effect (SME) for the first time. Rahmatabadi Davood et al. [23] successfully 3D-printed PVC samples using different printing parameters (including speed, grating angle, nozzle diameter and layer thickness) and studied their mechanical properties in compression, bending and stretching modes. The study shows that raster angle and printing velocity influence the mechanical properties significantly, whereas the layer thickness and nozzle diameter have little effect. The maximum tensile strength of 88.55 MPa is achieved, which implies the superiority of 3D-printed PVC mechanical properties compared to other commercial filaments. This study opens an avenue to additively manufacture PVC, which is the second most-consumed polymer with cost-effective and high-strength features. Moradi M et al. [24] conducted additive manufacturing of nylon through fused deposition modeling and studied the effects of process parameters such as layer thickness, filling rate, number of contours on maximum failure load, part weight, elongation at break and molding time. According to the calculation results, it is found that the layer thickness is an important principal variable for all responses.

In this paper, the preparation process of the liner is combined with 3D-printing technology to explore the application prospect of a 3D-printed liner in the front damage element of a tandem warhead. Firstly, the mechanical properties test samples of PLA and PLA-Cu materials were prepared by a 3D printer, and the solid high-pressure equation of state of the two materials was studied by shock wave compression technology. Then, the obtained high-pressure equation of state parameters was combined with the finite element simulation software to numerically simulate the jet-forming process of the 3D-printed liner, and the jet shape pulse X-ray shooting test was carried out to verify the numerical simulation results.

## 2. Materials and Research Method

The Ultimaker S5 3D printer from the Ultimaker company in the Netherlands was selected, and the forming method was fused deposition modeling (FDM). The 3D printer integrates accuracy and speed. The maximum size of the printed specimen can reach 330 × 240 × 300 mm, which meets the design requirements of the specimen required in this paper. Before printing, the sample first needs to establish a solid model in the three-dimensional software Solid Works according to the designed model style and size; then, save it and export it as an STL file, and import this file into the open-source slicing machine software Cura for Ultimaker S5 type 3D printer. Slice and print parameter settings, and finally, send the model of the completed slice to the 3D printer to complete the preparation of the sample.

**Figure 1:**
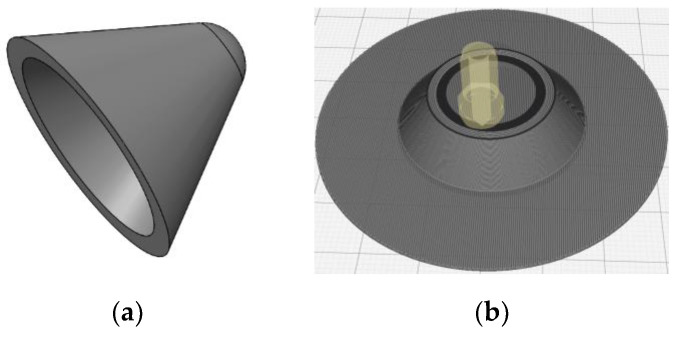
Preparation of liner: (**a**) Solidworks model. (**b**) Slicing method in Cura software.

**Figure 2:**
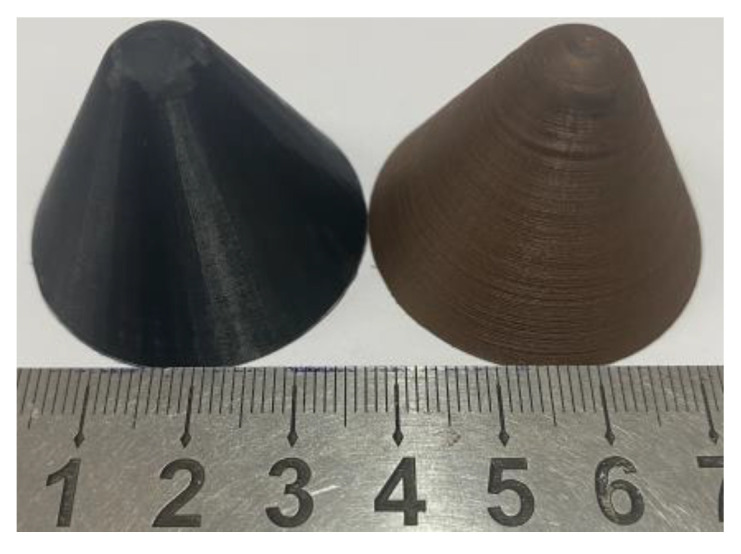
Liner physical diagram.

Before starting to print, you also need to enter the printing parameters in the 3D printer. In this paper, the filling density of 3D printing was selected to be 100%, and the internal voids were reduced as much as possible. Because the printing melting temperature of PLA material is about 190–230 °C, the temperature of the printer nozzle was set to 210 °C. At the same time, the temperature of the printing support platform was set to 60 °C so as to ensure that the specimen can be firmly pasted on the support platform without warping during the printing process. The fixed parameters are shown in Table 1. All specimens were printed at room temperature, and the printer was insulated during the printing process. Figure 1 and Figure 2 shows the preparation process of the liner and the physical diagram.

The materials used in this paper are PLA and PLA-Cu wire rods with a diameter of 2.85 mm. The manufacturer is Ultimaker. For commercial confidentiality, the manufacturer only provides the basic parameters of PLA wire rods, as shown in Table 2.

Several PLA- and PLA-Cu-shaped charge liners were prepared by 3D printing. After the shaped charge liner was printed, the electronic balance was used to weigh the shaped charge liner, and the true average mass and average density of the two materials were obtained, as shown in Table 3.

When modeling in three-dimensional software, the theoretical volume of the liner can be directly read out to be 5218.6 mm^3^, and the theoretical mass of the PLA liner can be obtained by the product of the liner volume and the density of the printed wire, which is 6.47 g. The real average mass of the PLA liner obtained by the electronic balance is 6.5 g, which shows that the error between the real mass and the theoretical mass is small. By calculating the true density of the liner, the average density of the PLA liner is 1.24 g/cm^3^, which is consistent with the density of the PLA wire, indicating that the liner obtained by the 3D-printing method in this paper has better compactness.

When using the same method, the real mass of PLA-Cu liner is 7.6 g, and the real density is 1.46 g/cm^3^, which is 17.74% higher than that of pure PLA liner. Due to technical protection, the merchant did not give the density and specific formula of PLA-Cu wire. In this paper, through the law of conservation of mass, combined with the copper material density of 8.96 g/cm^3^, the density of PLA-Cu material wire was calculated to be about 1.485 g/cm^3^, and then the content of Cu material in PLA-Cu wire was calculated to be about 2.85%.

**Figure 3:**
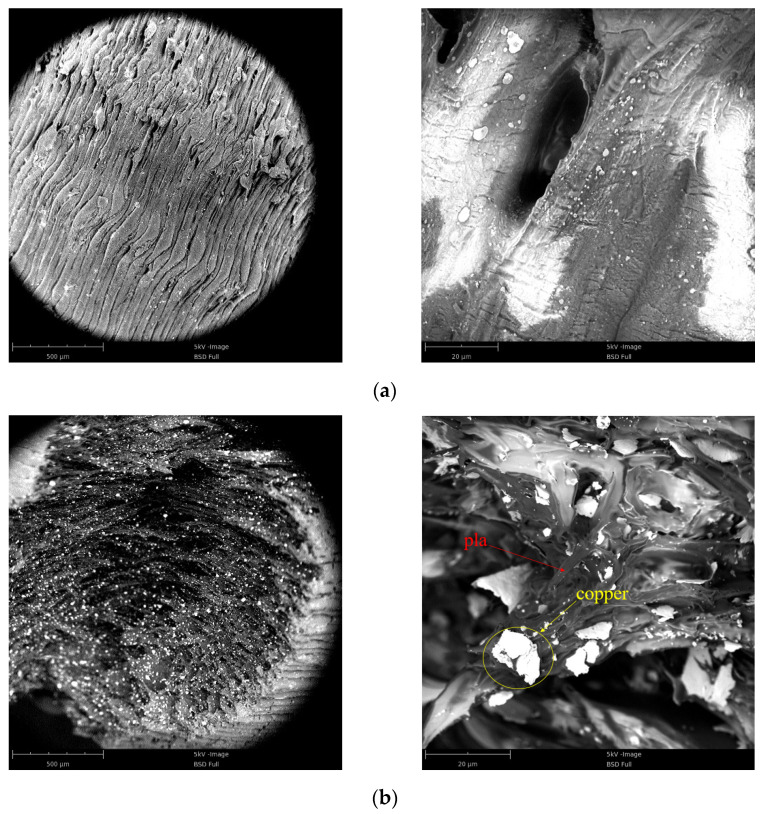
Scanning electron microscopy of specimen: (**a**) SEM images of PLA samples. (**b**) SEM images of PLA-Cu samples.

Figure 3a shows the SEM images of the PLA sample. It can be seen that the internal voids between each layer of material caused by the 3D-printing process are completely fitted. Figure 3b shows the SEM image of the PLA-Cu sample. It can be seen that the white copper material is an irregular block filler, which is evenly distributed in the PLA matrix, and the filler size is between 5 and 20 μm. PLA matrix has good adhesion. It can be seen from Figure 3 that the 3D-printed sample has good compactness.

## 3. Experimental Study on High-Pressure Equation of State

In the conventional mechanical system, there are generally only two independent thermodynamic variables. Starting from the same initial state, after different intensity shock wave compression, the thermodynamic state set that the medium can reach is expressed as a curve on any two independent thermodynamic variable planes, which is called the shock insulation line of the medium, that is, the Hugoniot line. In addition to following the universally applicable conservation law, the shock Hugoniot line reflects the relationship between the shock wave and the thermodynamic parameters of a specific material, which is a reflection of the dynamic characteristics of the material. Therefore, the measurement of the Hugoniot curve is the basis for the study of the high-pressure equation of state and other properties of materials.

Under high pressure, the anti-distortion ability of solid materials can often be ignored; that is, the shear strength of the material is not considered, so the distortion law part of the material constitutive can be ignored, and only the volume change law part is considered. At this time, the constitutive relationship is simplified to the relationship between hydrostatic pressure *P* and specific volume *V*, which is called the solid high-pressure equation of state. The main research content of this section is to study the solid high-pressure equation of state of 3D-printed PLA and PLA-Cu materials by symmetrical collision test.

The shock jump condition, together with the internal energy equation of state, consists of four equations, including five unknown parameters, *P*, *V*, *E*, *u* and *D*. The relationship between any two parameters is called the shock insulation line. There are three most commonly used: *P*-*V* line, *P*-*u* line and *D*-*u* line. Since various forms of shock insulation lines should satisfy the three conservation relations and internal energy equation of states on the shock wave front, different forms of shock insulation lines are not independent of each other but can be converted to each other. From the point of view of shock wave experimental research and testing technology, the parameters of velocity dimension, such as particle velocity *u* and shock wave velocity *D*, are generally easier to measure because they can be attributed to the measurement of distance Δ*S* and corresponding time interval Δ*t*, which is easier to achieve accurate measurement with current testing technology. The pressure, specific volume and temperature under dynamic conditions are relatively difficult to measure. Hugoniot lines are often used and measured in the form of *D-u* Hugoniot lines for high-pressure equation of states of materials. Therefore, at present, the measurement technology of shock wave velocity *D* and particle velocity *u* is relatively mature, and the accuracy of measurement is relatively high. This paper also mainly discusses the *D-u* form of shock insulation line.

For the same material, the Hugoniot line in the form of *D-u* is unique. A large number of studies have shown that for many solid materials, especially in the medium-pressure region, the *D-u* curve satisfies a linear relationship:

(1) $$D-u_{0}=C_{0}+S(u-u_{0})$$

In Equation (1), *C*_0_ and *S* are material constants.

It can be seen from Equation (1) that when *u* approaches 0, the non-isentropic shock wave approaches the isentropic sound wave, so *C*_0_ should represent the sound velocity of the material.

The experimental measurement of the *D-u* shock insulation line is to determine the relationship between the shock wave velocity *D* and the particle velocity *u* of the material to be tested or to determine the relationship between the shock wave velocity *D* and the shock wave pressure *P*, and then obtain the particle velocity through the conservation law. The former is called the absolute measurement method, and the latter is called the comparative measurement method. Because there is no need to introduce any assumption in the absolute measurement method, the thermodynamic parameters such as pressure, specific volume and specific internal energy of the Hugoniot state after the shock wave can be completely determined according to the conservation equation. Therefore, this paper used the absolute measurement method to obtain the Hugoniot line in the form of *D-u* of 3D-printed PLA and PLA-Cu materials.

**Figure 4:**
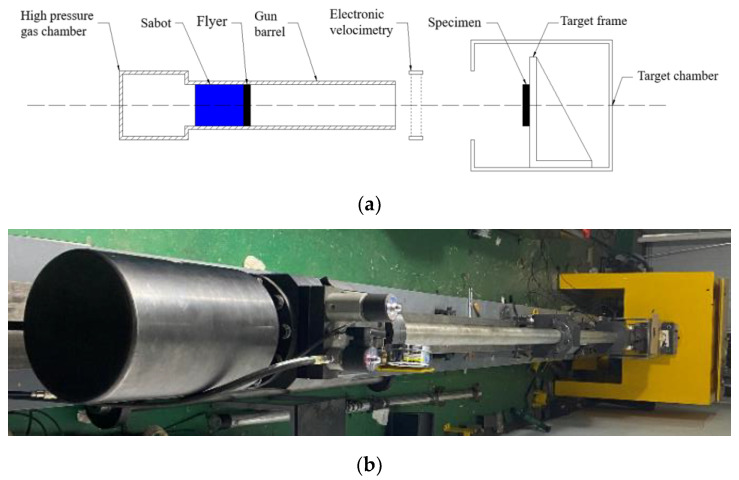
Structure diagram and physical diagram of one-stage light gas gun: (**a**) Structure diagram. (**b**) Physical diagram.

In the measurement of *D* and *u*, *D* is generally measured directly, while *u* is generally measured indirectly by the interaction of shock wave propagation through the flyer impacting the sample. In this paper, the Hugoniot line of 3D-printed PLA and PLA-Cu materials in the form of *D-u* was measured by using the one-stage light gas gun experiment system. The structure diagram and physical diagram of the light gas gun are shown in Figure 4, and its diameter is 30 mm.

**Figure 5:**
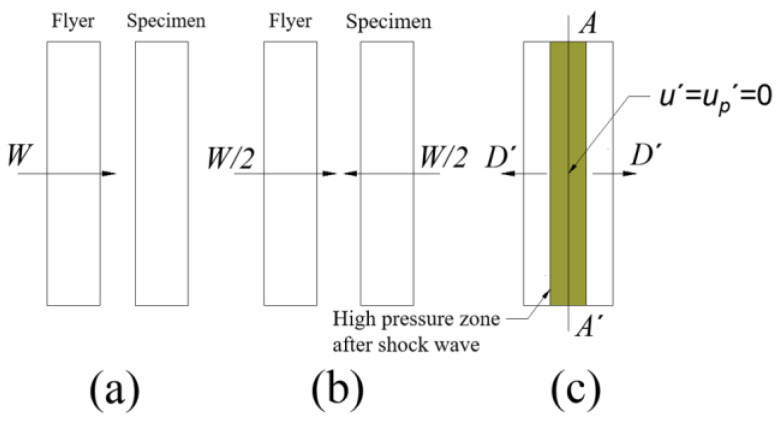
Flyer impact test sample with velocity of *W* motion in laboratory coordinate system: (**a**) Images in the test coordinate system. (**b**) The image before the flyer hits the target plate in a coordinate system moving at a speed of *W*/2 (relative motion coordinate system). (**c**) Shock wave motion image after hitting the target plate.

During the test process, by changing the pressure of the high-pressure gas chamber, the sabot and the flyer are given different impact velocities to impact the sample to be tested at the center of the target frame, and the projectile velocity is obtained using an electronic velocimetry. In the absolute method measurement, the flyer is required to be completely equal to the material to be tested. According to the symmetry principle, after the flyer impacts the target plate, the impact compression state of the two is exactly the same, so this test is also called the “symmetrical collision test”. The schematic diagram of the flyer impacting the target plate is shown in Figure 5. *W* is the velocity of the flyer, and *D*′ is the velocity of the shock wave measured in the coordinate system moving at the speed of *W*/2. It is assumed that the flyer collides the static sample to be tested at a speed *W*. If the collision process (relative motion coordinate system) is observed in a coordinate system moving at a speed of *W*/2 relative to the laboratory coordinate system, the observer in the motion coordinate system will see as shown in Figure 5c. At the moment of collision, two symmetrical shock waves with reverse motion are formed on both sides of the collision surface *AA*′, and the collision interface *AA*′ remains static so that the velocity of the material on both sides of the interface layer at the moment of collision *u*′ = 0. The velocity *u*′ of the interface layer material is equal to the particle velocity *u_p_*′ after the two shock waves, and the particles after the two symmetrical shock waves of the reverse motion will also remain stationary, *u_p_*′ = *u*′ = 0.

Because the target plate is thin, it can be considered that the shock wave propagates steadily in it. At the same time, because of the symmetrical collision, the material of the flyer and the target plate is exactly the same. According to the principle of relative motion, the particle velocity *u_p_* measured by the observer in the laboratory coordinate system should be equal to the sum of the motion velocity *W*/2 measured in the coordinate system and the particle velocity *u*′ measured in the relative motion coordinate system; that is, *u_p_* = *W*/2 + *u*′ = *W*/2. Therefore, in the symmetric impact test, the particle velocity after the wave observed in the laboratory coordinate system is equal to half of the flyer velocity.

**Figure 6:**
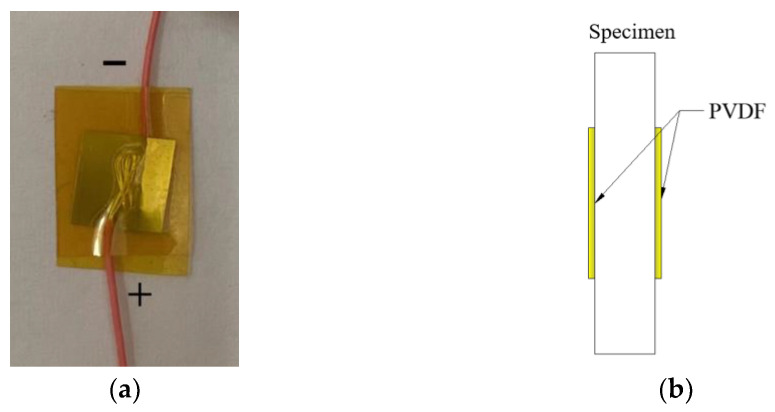
Physical diagram and paste diagram of PVDF pressure sensor: (**a**) Physical diagram. (**b**) Paste diagram.

The PVDF pressure sensor is used to measure the propagation signal of the shock wave in the sample. Figure 6 shows the physical diagram of the PVDF pressure sensor and the paste diagram on the sample. Before the test, the PVDF pressure sensor is first pasted on both ends of the sample thickness direction, and the take-off time difference ∆*t* of the two sensors is collected by an oscilloscope. The shock wave velocity *D* can be obtained by dividing the sample thickness by the shock wave propagation time difference. By giving the light gas gun system different sizes of gas chamber pressure, the flyer can hit the target plate at different speeds, and a series of *D* and *u* parameters are obtained. By analyzing and fitting them, the Hugoniot relationship in the form of *D-u* of two materials can be obtained.

**Figure 7:**
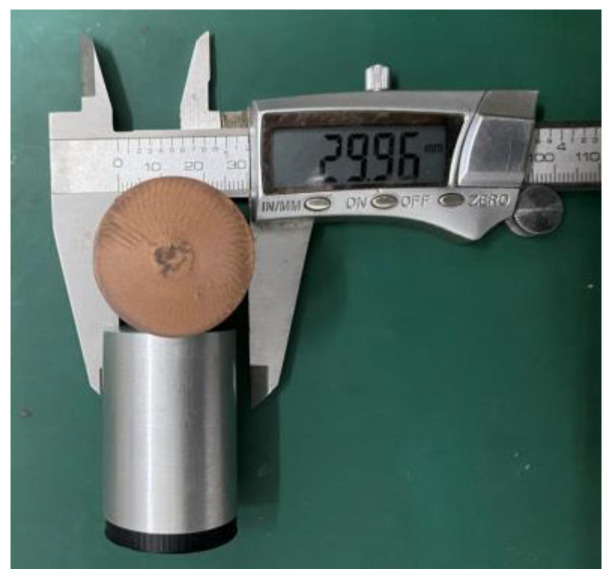
Sabot and flyer.

The diameter of the flyer and the sample to be tested is consistent with the diameter of the one-stage light gas gun. The diameter of the flyer *d_F_* and the diameter of the sample to be tested *d_S_* are both 30 mm. The subscripts “*F*” and “*S*” represent the flyer and the sample to be tested, respectively. The thickness is 4 mm, and the width-to-thickness ratio of the sample is 7.5, which meets the requirement of greater than 2. At the same time, due to the requirements of the absolute measurement method, the size of the sample to be tested is exactly the same as that of the flyer. In addition, during the test, the flyer and the sabot are bonded with 502 strong adhesives, as shown in Figure 7, to ensure that the flyer does not fall from the sabot during the test.

**Figure 8:**
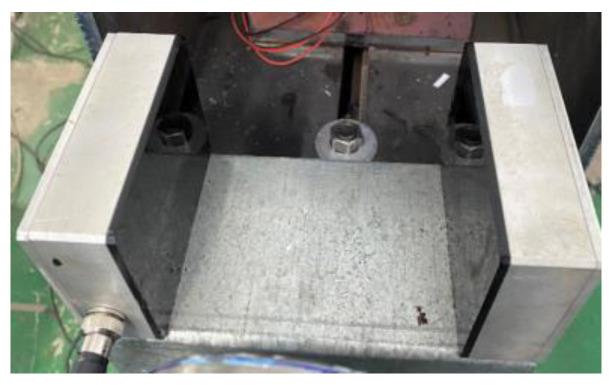
Electronic velocimeter.

**Figure 9:**
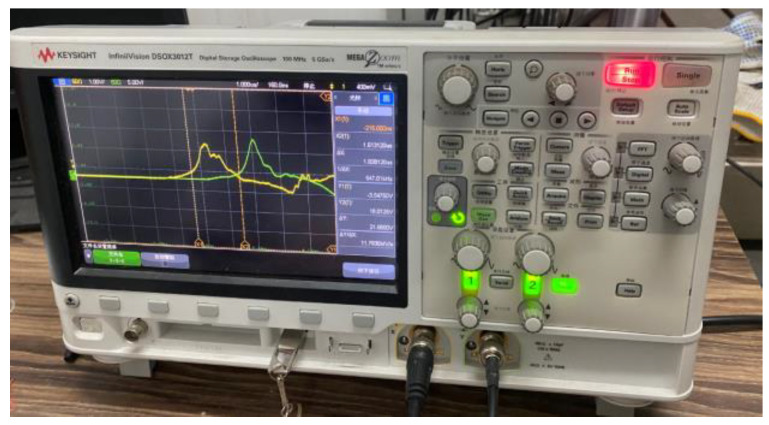
Typical test curves recorded by the oscilloscope.

By changing the pressure value of the high-pressure chamber, the flyer is given different speeds to impact the sample to be tested, and the movement time of the shock wave in the sample to be tested under a series of impact velocities is obtained by electronic velocimeter in Figure 8. Figure 9 is the typical test curve recorded by the oscilloscope during the test. It can be seen that the test curve is relatively smooth, and the take-off time is obvious. Table 4 is the test result.

**Figure 10:**
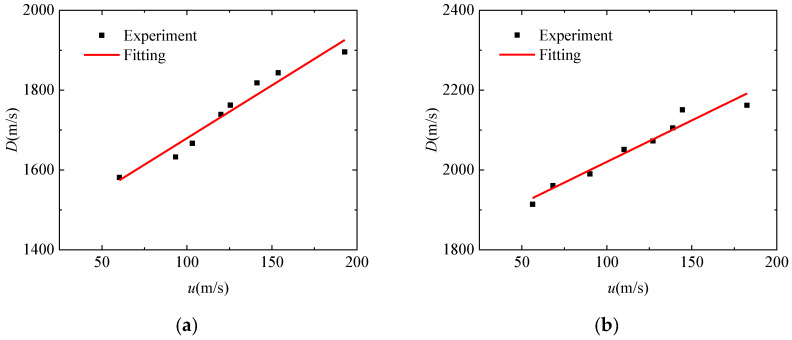
*D*-*u* fitting curve: (**a**) PLA. (**b**) PLA-Cu.

According to Equation (1), the *D-u* equation is fitted. The results are shown in Figure 10. It can be seen that the *D-u* curves of the two materials show a linear relationship, and the test results are in good agreement with the fitting results.

The *D-u* equation fitted by PLA material is:

(2) D=2.65u+1414.44

The *D-u* equation fitted by PLA-Cu material is:

(3) D=2.08u+1812.48

When studying the propagation of shock waves in solids under high pressure, the Grüneisen equation is the most commonly used equation of state, and its general form is:

(4) $$P=P_{k}\left(V\right)+\frac{\gamma \left(V\right)}{V}\left[E-E_{k}\left(V\right)\right]$$

In Equation (4), $P_{k}\left(V\right)$ is the cold pressure, $E_{k}\left(V\right)$ is the cold energy, and $\gamma \left(V\right)$ is the Grüneisen coefficient. 

In the case of ignoring the distortion of the material, the simple polynomial Grüneisen equation can be used to describe the capacitive part of the material.

(5) $$P=C_{1}\mu +C_{2}\mu^{2}+C_{3}\mu^{3}$$

In Equation (5), *μ* is the compression degree of the material, which is defined as:

(6) $$\mu =\frac{\rho }{\rho_{0}}-1$$

In Equation (6), *ρ* is the actual density of the material, and *ρ*_0_ is the initial density of the material. 

In the symmetrical collision test, the compressibility of 3D-printed PLA and PLA-Cu materials is regarded as a small amount. The flyer follows the laws of mass conservation, momentum conservation and energy conservation during the impact of the sample to be tested. The basic equation is:

(7) $$\rho_{0}\left(U-u_{0}\right)=\rho (U-u)$$

(8) $${P-P}_{0}=\rho_{0}\left(U-u_{0}\right)\left(u-u_{0}\right)$$

(9) $${E-E}_{0}=\frac{1}{2}\left(P-P_{0}\right)\left(\frac{1}{\rho_{0}}-\frac{1}{\rho }\right)$$

Simultaneous Equations (5)–(7) are available:

(10) $$u=\frac{c_{0}\mu }{1-(S-1)\mu }$$

The analysis shows that there is 0 < (S−1)μ < 1, which can be obtained by Taylor expansion:

(11) $$u=c_{0}\mu [1+\left(S-1\right)\mu +{\left(S-1\right)}^{2}\mu^{2}+\ldots ]$$

In the symmetrical collision test, there is also:

(12) $$P_{F}=P_{S}=P$$

(13) $$u_{F}=u_{S}=u$$

Simultaneous Equations (8) and (11)–(13) are available:

(14) $$P=\rho_{0}C_{0}^{2}[\mu +\left(2S-1\right)\mu^{2}+\left(3S^{2}-4S+1\right)\mu^{3}+\ldots ]$$

The higher-order terms after $\mu^{3}$ are omitted, and the simultaneous Equations (5) and (14) are available:

(15) $$C_{1}=\rho_{0}C_{0}^{2}$$

(16) $$C_{2}=\rho_{0}C_{0}^{2}\left(2S-1\right)$$

(17) $$C_{3}=\rho_{0}C_{0}^{2}\left(3S^{2}-4S+1\right)$$

The polynomial Grüneisen high-pressure state equations of PLA and PLA-Cu materials can be obtained by taking the parameters of Equations (5) and (6) into the above equations:

PLA:

(18) $$P=1.03\mu +16.92\mu^{2}+192.79\mu^{3}$$

PLA-Cu:

(19) $$P=1.17\mu +17.3\mu^{2}+215.57\mu^{3}$$

When the *D-u* shock insulation line of the material shows a linear relationship, the slope *S* of the linear equation has a direct relationship with the Grüneisen coefficient $\gamma \left(V\right)$:

(20) $$S=\frac{1}{2}\;\gamma \left(V\right)+\frac{3}{2}$$

According to Equation (20), the Grüneisen coefficients of PLA and PLA-Cu materials are 2.3 and 1.16, respectively.

## 4. Numerical Simulation and Experimental Verification of Jet Forming

**Figure 11:**
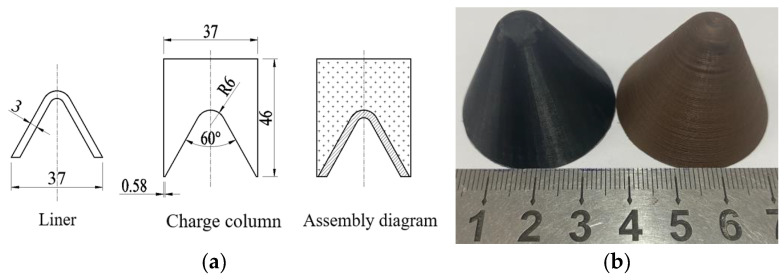
Preparation of liner: (**a**) Size of the liner and the structure of the warhead. (**b**) Physical diagram of liners.

The front charge of the tandem warhead produces detonation waves and detonation products after explosion. In order to reduce the impact on the rear warhead, the volume of the warhead should be reduced as much as possible while ensuring the damage power of the front warhead. Therefore, the front stage of the tandem warhead is designed as a small-caliber-shaped charge in this paper. The caliber is 37 mm, the wall thickness of the liner is 3 mm, and the cone angle is 60°. The size of the liner and the structure of the warhead are shown in Figure 11a. Figure 11b is the physical diagram of PLA and PLA-Cu liners. After the printing, the edge and top of the liner are polished with sandpaper to ensure the fit with the grain during the test.

### 4.1. Numerical Simulation of Jet Forming

The liner under the action of detonation load involves the problem of large deformation of the material. Therefore, the Arbitrary Lagrangian Eulerian (ALE) algorithm is often used to study the calculation of shaped charge jet forming. The algorithm only divides the space into grids in advance. Because the grid is fixed in the space and the deformed object passes through it, the problem of large distortion of the grid distortion can be solved, but it is difficult to deal with the interface of different objects.

The Smoothed Particle Hydrodynamics (SPH) algorithm is a meshless Lagrangian algorithm. There is no problem of mesh element distortion or failure in the calculation process. It has obvious advantages in simulating extreme deformation and failure types such as hypervelocity collision and target penetration. The SPH algorithm can accurately simulate the head expansion effect of the polymer jet, and the numerical simulation of the jet shape is highly consistent with the results of the polymer jet pulse X-ray shooting. Therefore, in this paper, the SPH calculation method was used in AUTODYN finite element software to carry out numerical simulation research on the jet forming of 3D-printed liners.

The material model is used to describe some special changes in the material under the action of load. In the numerical simulation, a reasonable material model and parameters can be selected to obtain accurate results. The definition of material in AUTODYN software is defined by equation of state, strength model and failure model, which can flexibly select the required material model according to the actual situation.

In the numerical simulation, the materials involved in this section are explosives and liners. The liner materials are 3D-printed PLA and PLA-Cu materials. The equation of state is the shock impact state equation, and the strength model is the Cowper–Symonds model. The explosive material is 8701 explosive, and the JWL equation of state is used to describe the detonation process of the explosive. The JWL equation of state parameters and C-J parameters are shown in Table 5.

In the table, *ρ is* the average density of the main charge prepared for the experiment, *D* is the detonation velocity, *E* is the energy density per unit volume, and *P_CJ_* is the detonation pressure of the explosive.

**Figure 12:**
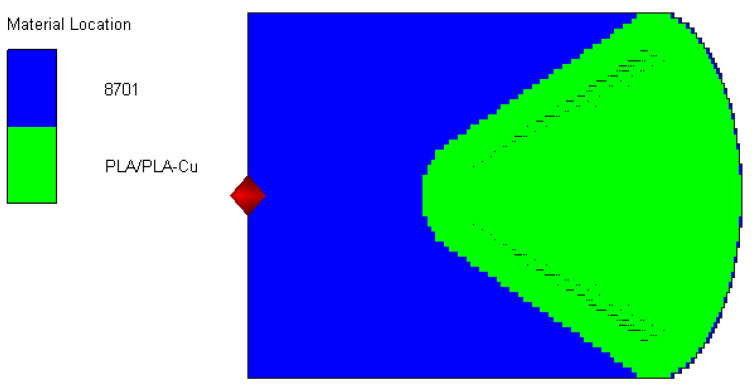
SPH algorithm: three-dimensional half-finite element model.

Figure 12 shows the finite element model of the SPH algorithm established in AUTODYN software. It is composed of explosive and liner particles. The detonation point is set at the center point of the bottom end face of the charge.

**Figure 13:**
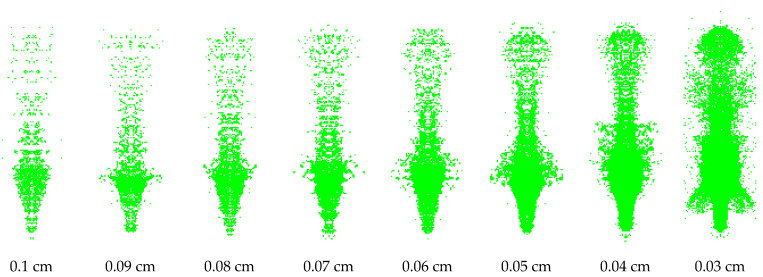
The effect of SPH particle gap on the PLA jet shape at 20 μs.

**Figure 14:**
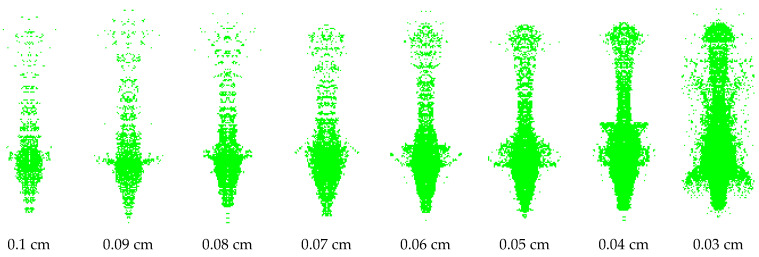
The effect of SPH particle gap on the PLA-Cu jet shape at 20 μs.

Because the SPH method represents the continuous material as a set of moving discrete particles with velocity in the calculation process, the particles follow the mass, momentum and energy conservation theorems and combine the calculation model and parameters given to the particle material to solve so as to obtain the motion law of the material. Therefore, the influence of particle gap on jet forming must be considered in the calculation process. Figure 13 and Figure 14 are the jet patterns of PLA and PLA-Cu liners at 20 μs under different SPH particle gaps in numerical simulation.

Figure 13 and Figure 14 show that both materials form a high-speed-shaped jet under different particle gaps. The particle gap has a significant effect on the formation of the jet. The higher the particle concentration, the better the continuity of the jet. When the particle gap is greater than 0.07 cm, the number of particles in the unit area of the jet is small, and the expansion degree of the head diameter is large, resulting in a serious fracture of the jet head and insufficient continuity. When the particle gap is greater than 0.05 cm, there are more scattered particles in the jet slug, which is not conducive to the overall formation of the jet. When comparing the jets with particle gaps of 0.06 cm and 0.05 cm, it is found that the jet formed by the latter was optimized in both morphology and continuity. Considering the time efficiency of numerical simulation, it is considered that 0.05 cm is the best choice for the SPH particle gap.

In the calculation process, it is found that the length and head velocity of the jet do not change with the change in SPH particle concentration. The average length of the PLA jet at 20 μs is 127.2 mm, and the average head velocity is 9232.5 m/s. The average length of the PLA-Cu jet at 20 s is 120.4 mm, and the average head velocity is 8590.75 m/s. When comparing the two jet shapes, it can be seen that the radial expansion degree of the PLA jet is greater than that of the PLA-Cu jet under the same SPH particle gap; that is, the cohesion of the PLA-Cu jet is better.

### 4.2. Pulse X-ray Shooting Experiment Verification of Jet Forming

In order to verify the validity of the numerical simulation results of the jet shape, the pulse X-ray photography system was used to carry out the jet shape shooting experiment of 3D-printed PLA and PLA-Cu liners. In this paper, the jet forming of two kinds of liner materials was analyzed from the jet shape. The characteristic parameters of the jet of the two materials were obtained, and the average head velocity of the jet was calculated.

The basic principle of pulse X-ray photography is that the X-ray tube generates X-rays through the discharge of a high-voltage power supply. When the X-ray beam projects an object, high-energy, short-wavelength rays can penetrate the object to a certain extent. The X-ray ability of the object depends on the energy of the radiation and the density and thickness of the object. Low-energy radiation is more easily absorbed than high-energy radiation, and objects with high density and thickness are more easily absorbed. By modulating the emitted X-ray beam, different object shapes can be rendered on the X-ray film.

**Figure 15:**
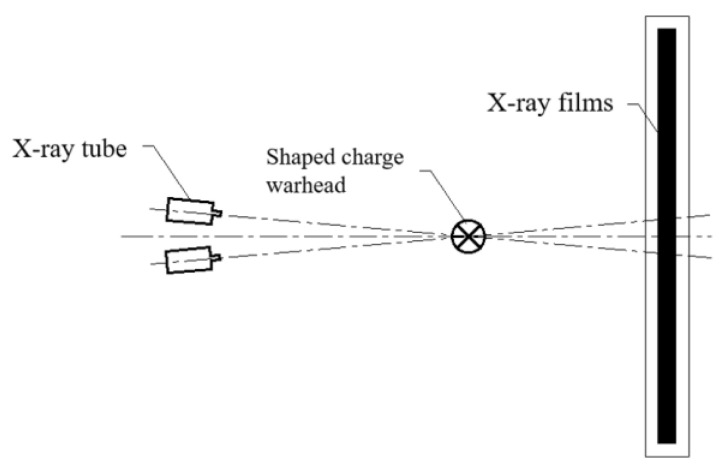
Principal diagram of pulse X-ray shooting.

The jet pulse X-ray shooting experiment used HP43737 type 300 KV pulse X-ray; the output current was 5 KA, and the pulse width was 50 ns. In this experiment, the basic principle of pulse X-ray photography is shown in Figure 15. The shaped charge warhead is kept perpendicular to the X-ray emission tube to ensure that the jet can be parallel to the film after forming while keeping the two X-ray tubes placed at a smaller intersection angle. By reasonably setting the different light output times of the two pulse X-ray machines, two X-ray images at different times can be obtained in one test. After processing, the jet shape at two times can be obtained. Finally, according to the position coordinates of the jet head in the two images, the length of the forward movement of the jet head in the two shooting intervals can be calculated, and then the average velocity of the jet head can be calculated by combining the difference between the two shooting times.

**Figure 16:**
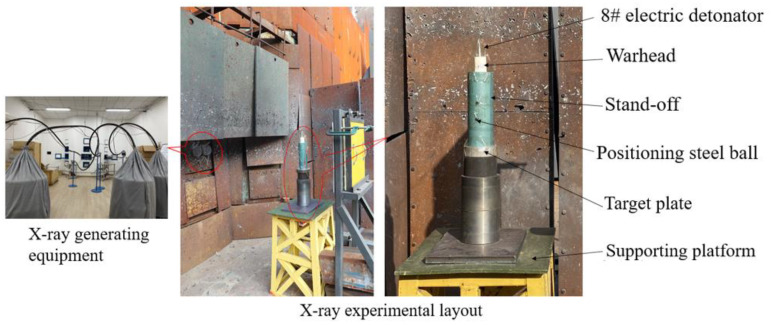
Jet X-ray shooting equipment and test site layout.

Figure 16 shows the pulse X-ray shooting equipment and test site layout of the shaped charge jet. The shaped charge was detonated by an 8# electric detonator. After detonation, two pulse X-ray machines emit light in turn according to the set delay time, leaving an image of the shaped jet on the film. In order to fully stretch the jet in the air, the blasting cylinder was designed to be six times the charge diameter, and the material was highland barley paper. At the same time, in order to facilitate the reading of the actual movement distance of the jet head between the two time differences on the X-ray photograph, two positioning steel balls with a diameter of 5 mm were pasted on the blaster before the test. As a scale, the actual distance between the two positioning steel balls is Y = 50 mm.

**Figure 17:**
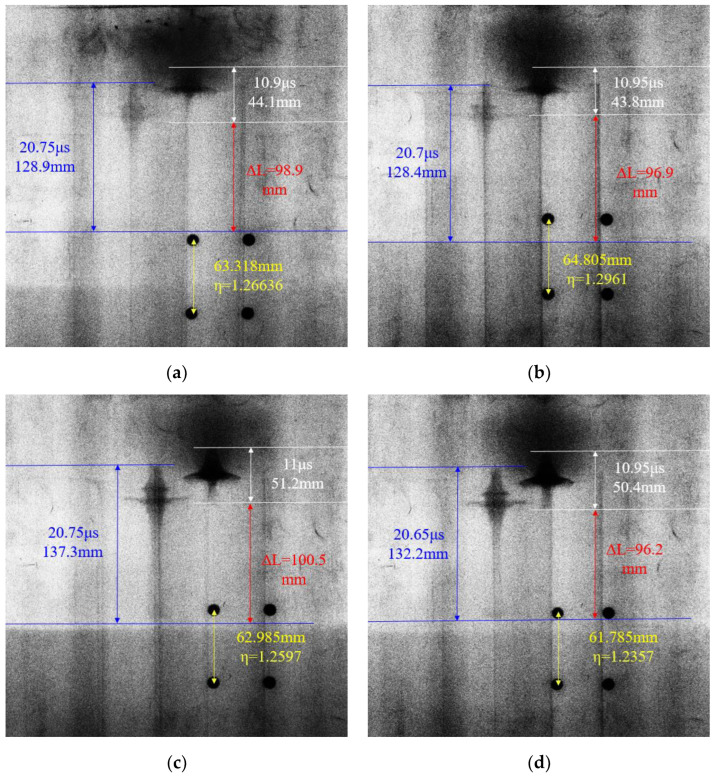
Pulse X-ray shooting test results: (**a**) PLA-1#. (**b**) PLA-2#. (**c**) PLA-Cu-1#. (**d**) PLA-Cu-2#.

Whether the ideal jet shape can be obtained is directly related to the selection of the time node of the two shots. If the preset time is too small or too large, the jet will not be fully stretched or directly hit the target plate. In this paper, the delay effect of pulse X-ray shooting equipment was considered. During the experiment, the preset time points T_1_ and T_2_ of the two flashes of the pulse X-ray machine were set to 10 μs and 20 μs, respectively. At the same time, considering the contingency of the experiment results, a set of parallel experiments was set up for the liners of each material to ensure the credibility of the test results. The experimental results of pulse X-ray photography of the jet shape are shown in Figure 17.

It can be seen from Figure 17 that both PLA and PLA-Cu liners form a shaped charge jet under the action of a detonation wave. The liner is basically completely crushed at T_1_, and the jet head is formed. It has a large head diameter and a divergent shape. The jet head material shows a clear trend of flying around. With the increase in time, the amplitude of the jet flying around also increases, showing obvious characteristics of “expanded particle flow”. At the same time, there are two “circles” with different diameters in the slug at the tail of the jet. The “collapse circle” forms when the liner material is crushed near the jet head. This occurs because, as the detonation wave reaches the bottom end of the liner due to sudden unloading, the fracture occurs at a distance of 0.5–1 mm from the bottom end of the liner, and the diameter of the slug at the tail of the jet is far from the jet head.

From the overall point of view of the jet at T_2_, the shadow part of the PLA jet is obviously shallower than the PLA-Cu jet. At this time, the head shape of the PLA jet is basically invisible, and only the pestle at the tail of the jet can be seen. The shape of the tail slug, neck and head of the PLA-Cu jet is clearer, and the contour is more obvious. The shape from the caving ring to the jet head is similar to a “hyperbolic” shape. Moreover, it can be seen from the X-ray photographs that there is a density gradient with a gradual decrease in the density of the jets of the two materials from the slug of the tail to the head of the jet.

At the end of the experiment, the distance *y* between the two positioning steel balls on the pulse X-ray image in each experiment was measured. According to Equation (21), the ratio of the length of the jet in the X-ray image to the real length can be calculated; that is, the scale *η*.

(21) $$\eta =\frac{y}{Y}$$

Secondly, the real time points t_1_ and t_2_ of the two shots are read out by the X-ray recording device, and the length $l_{t_{1}}$ and $l_{t_{2}}$ of the jet at the two time points in the X-ray pictures are measured at the same time. The real length of the jet is equal to the length of the X-ray picture divided by the scale *η*. The calculation results of the characteristic parameters and the real length of the jet captured by pulse X-ray are shown in Table 6.

In Table 6, Δ*t* = *t*_2_ − *t*_1_, $L_{t_{1}}$ and $L_{t_{2}}$ are the real lengths of the jets corresponding to *t*_1_ and *t*_2_, respectively. From the recorded real time point, it can be seen that the delay time of the pulse X-ray shooting equipment is basically between 0.65 and 1 μs, and the time difference between the two-output light is between 9.7 and 9.85 μs.

According to the calculation results in Table 6, in the two parallel experiments, the length of the PLA jet at *t*_1_ is 34.8 mm and 33.8 mm, and the length at *t*_2_ is 101.8 mm and 99.1 mm. The length of the PLA-Cu jet at *t*_1_ is 40.6 mm and 40.8 mm, and the length at *t*_2_ is 109.0 mm and 107.0 mm. It can be seen that the error of the two parallel experiment results is small, and the repeatability is good. The jet length and shape at the same time point are basically the same, which proves that the experiment results are reliable and stable.

In Figure 17, the distance Δ*L* of the jet head at two time points on each X-ray photo is measured, and the average velocity $\bar{v}$ of the jet head in this stage can be calculated. Because there is a velocity gradient in the jet itself, in the process of rapid movement of the jet head, the slug of the jet is also moving forward relatively slowly. Therefore, it should be noted that the jet head distance difference Δ*L* is not equal to the difference in the length of the jet at two time points. Equation (22) is the calculation formula for the average velocity of the jet head.

(22) $$\bar{v}=\frac{\Delta L}{\eta \left(t_{2}-t_{1}\right)}$$

The calculation results of the average velocity of the head of the jet are shown in Table 7. It can be seen that the average velocity of the head of the PLA jet at *t*_1_ − *t*_2_ is 7798.35 m/s, and the average velocity of the head of the PLA-Cu jet is 8104.25 m/s.

**Figure 18:**
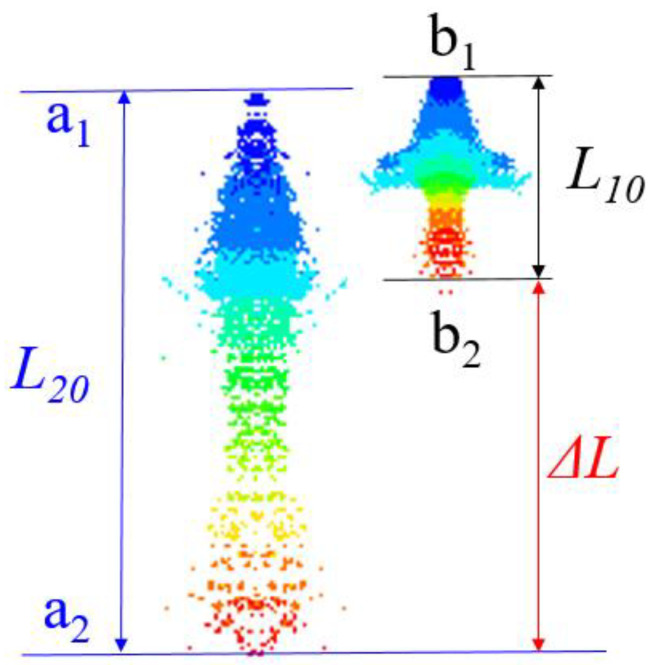
Numerical simulation of jet average velocity calculation diagram.

When comparing the numerical simulation results with the experimental results, it can be seen that the morphology of the numerical simulation jet is similar to the experimental results, which shows that it is feasible to use the SPH method to simulate the jet-forming process of the 3D-printed liner. However, the length and average velocity of the numerical simulation jet are different from the experimental results. The average velocity $\bar{v}$ (Δ*L*/10) of the simulated jet at two moments is obtained by using the same calculation method as the experiment, as shown in Figure 18.

The average velocity of the numerical simulation PLA jet is 8840 m/s, while the experimental results show that the real average velocity of the PLA jet is 7798.35 m/s, and the two have obvious differences. There are two reasons for the large error.

One is that the numerical simulation is an ideal environment, ignoring the influence of human operation and test environment in the experiment process, resulting in the average velocity of the numerical simulation jet being higher than the test value.

The second reason is that for the PLA liner under the action of explosives, the jet head produced a certain gasification. The density of PLA material is lower than that of PLA-Cu material. According to the conservation of energy, it is reasonable to obtain a higher head velocity than a PLA-Cu jet under the same charge structure. However, from the experimental results, the average velocity of the PLA-Cu jet is higher than that of the PLA jet. This phenomenon is caused by the fact that the 3D-printed pure PLA liner is a polymer material, and part of the polymer material is vaporized under the action of high temperature and high-pressure detonation products, resulting in the length of the PLA jet on the pulse X-ray photography. The length of the jet is shorter than the length of the numerical simulation jet, so the average velocity of the jet is lower.

**Figure 19:**
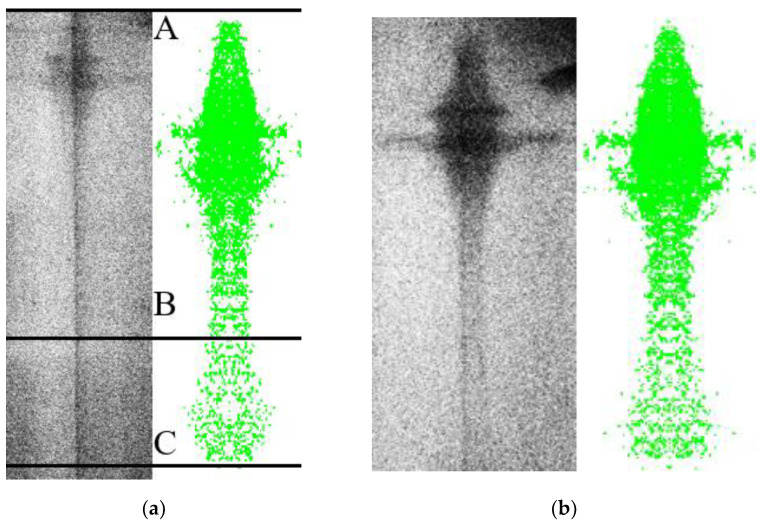
Comparison of numerical simulation and experimental results: (**a**) PLA jet morphology. (**b**) PLA-Cu jet morphology.

Figure 19 is the comparison between the numerical simulation jet and the experimental results at 20 μs. The AB section of the PLA jet is the true length of the PLA jet, and the BC section is the PLA jet gasification section. In the numerical simulation, the length of the PLA jet at 20 μs is 127.2 mm, and the average length of the PLA jet at 20 μs in the pulse X-ray shooting experiment is 100.45 mm, indicating that the length of the gasification part of the PLA jet accounts for about 21% of the length of the numerical simulation jet. 

The average velocity of the PLA-Cu jet in the numerical simulation is 8090 m/s, and the average velocity in the experiment is 8104.25 m/s. The error between the two is small. Moreover, the length of the PLA-Cu jet in the numerical simulation is also very close to the length of the jet in the X-ray photograph, which proves the validity of the numerical simulation results of jet forming and also proves the correctness of the calculation of the proportion of PLA jet gasification in this paper.

## 5. Results

In this paper, the samples and liners of polylactic acid (PLA) material and copper powder-filled polylactic acid (PLA-Cu) material were prepared by a 3D printer. Based on the one-stage light gas gun test system, the Grüneisen equation of state parameters of the two materials was obtained by a symmetrical collision experiment. Then, the parameters of the high-pressure equation of state were combined with the finite element simulation software to numerically simulate the jet-forming process of the 3D-printed liner. Based on this research, the jet shape pulse X-ray shooting experiment of the 3D-printed liner was carried out. The main conclusions are as follows:

(1) The *D-u* impact insulation lines of 3D-printed PLA and PLA-Cu materials were obtained by symmetrical collision experiment. Based on this, the polynomial Grüneisen high-pressure equation of state and state coefficient of the two materials were obtained by theoretical calculation, which provided model data support for numerical simulation of jet forming and penetration of 3D-printed liners.

(2) The SPH finite element simulation method was used to calculate the jet-forming process of 3D-printed PLA and PLA-Cu liners. It was found that the jet of these two materials showed obvious characteristics of “expansion particle flow”, and the shape of the high-speed jet part was similar to a “hyperbola”. With the increase in time, the amplitude of the jet flying around also increases. The real shape of two kinds of material jets was obtained by pulse X-ray photography. Firstly, the real characteristic parameters of the jets were obtained. Secondly, it was found that the two kinds of jets not only had a velocity gradient from the tail of the slug to the head of the jet but also had a gradually decreasing density gradient.

(3) When comparing the X-ray images of the jet shape at 20 μs, it was found that the shape of the slug, neck and head of the PLA-Cu jet is clearer, and the contour is more obvious. However, the PLA jet can only see the shape of the tail and the general position of the head, and the complete shape of the PLA jet cannot be seen. Based on the numerical simulation results and theoretical analysis, it is considered that a part of the 3D-printed pure PLA liner is vaporized under the action of high temperature and high-pressure detonation products, which makes it impossible to see the complete shape of the PLA jet on the pulse X-ray photographs, while the PLA-Cu liner makes the pulse X-ray photographs more obvious because of the existence of copper powder material. At the same time, the length of the PLA jet is calculated by comparing the length of the PLA jet taken by numerical simulation and pulse X-ray, and the length of the gasification part in the PLA jet is calculated to be about 21% of the total length of the PLA jet.

## Figures and Tables

**Table 1:** Three-dimensional printing fixed parameters.

Parameter	Nozzle Parameter (mm)	Printing Speed (mm/min)	Layer Height (mm)
Numerical Value	0.4	20	0.01

**Table 2:** Physical properties of PLA wire.

Attribute	Testing Method	Unit	Test Condition	Typical Value
Density	ASTM D792	g/cm^3^	-	1.24
The melt flow rate	ASTM D1238	g/10 min	210 °C, 2.16 kg	7
Melt density	-	g/cm^3^	230 °C	1.08
Melting point	-	°C	-	155–170

**Table 3:** Measurement results of mass and density of 3D-printed liner.

Materials	Mass (g)	Density (g/cm^3^)
PLA	6.5 (±0.1)	1.24 (±0.02)
PLA-Cu	7.6 (±0.2)	1.46 (±0.03)

**Table 4:** Symmetrical impact test data.

Material	*W* (m/s)	*u* (m/s)	Δ*t* (μs)	*D* (m/s)	Material	*W* (m/s)	*U* (m/s)	Δ*t* (μs)	*D* (m/s)
PLA	112.76	56.38	3.35	1194.03	PLA-Cu	120.48	60.24	2.585	1547.39
	136.59	68.30	2.59	1544.40		186.65	93.31	2.45	1632.65
	181.08	90.05	2.15	1860.47		206.40	103.20	2.59	1544.40
	220.26	110.13	2.03	1970.44		239.95	119.98	2.30	1739.13
	254.29	127.15	1.98	2020.20		250.94	125.47	2.65	1509.43
	277.58	138.79	1.90	2105.26		282.29	141.15	1.89	2116.40
	288.91	144.46	1.86	2150.54		307.34	153.67	1.77	2259.89
	364.96	182.48	1.65	2424.24		385.54	192.77	1.51	2649.01

**Table 5:** JWL equation of state parameters and C-J parameters of 8701 explosive.

*A* (GPa)	*B* (GPa)	*R_1_*	*R_2_*	*W*	*ρ* (g/cm^3^)	*D* (m/s)	*E* (KJ/m^3^)	*P_CJ_* (GPa)
854.5	20.493	4.6	1.35	0.25	1.71	8315	8.5 e6	29.5

**Table 6:** The characteristic parameters of pulse X-ray jet and the calculation results of real jet length.

Experiment	*y* (mm)	*η*	*t*_1_ (μs)	*t*_2_ (μs)	Δ*t* (μs)	$l_{t_{1}}$ (mm)	$l_{t_{2}}$ (mm)	$L_{t_{1}}$ (mm)	$L_{t_{2}}$ (mm)
PLA-1#	63.318	1.26636	10.90	20.75	9.85	44.1	128.9	34.8	101.8
PLA-2#	64.805	1.2961	10.95	20.70	9.75	43.8	128.4	33.8	99.1
PLA-Cu-1#	62.985	1.2597	11.00	20.75	9.75	51.2	137.3	40.6	109.0
PLA-Cu-2#	61.785	1.2357	10.95	20.65	9.7	50.4	132.2	40.8	107.0

**Table 7:** Calculation results of average velocity of jet head.

Experiment	Δ*L* (mm)	$\bar{v}$ (m/s)
PLA-1#	98.9	7928.7
PLA-2#	96.9	7668.0
Average	97.9	7798.35
PLA-Cu-1#	100.5	8182.7
PLA-Cu-2#	96.2	8025.8
Average	98.35	8104.25

## Data Availability

Not applicable.
